# Supplementary material for: Inhaled Nitric Oxide Promotes Angiogenesis in the Rodent Developing Brain
Source: Int J Mol Sci. 2023 Mar 20;24(6):5871. doi: 10.3390/ijms24065871 (PMC10054632; doi:10.3390/ijms24065871)
Supplement: Supplementary file 1 [file ijms-24-05871-s001.zip › ijms-2211280-supplementary.pptx]

## Slide 1
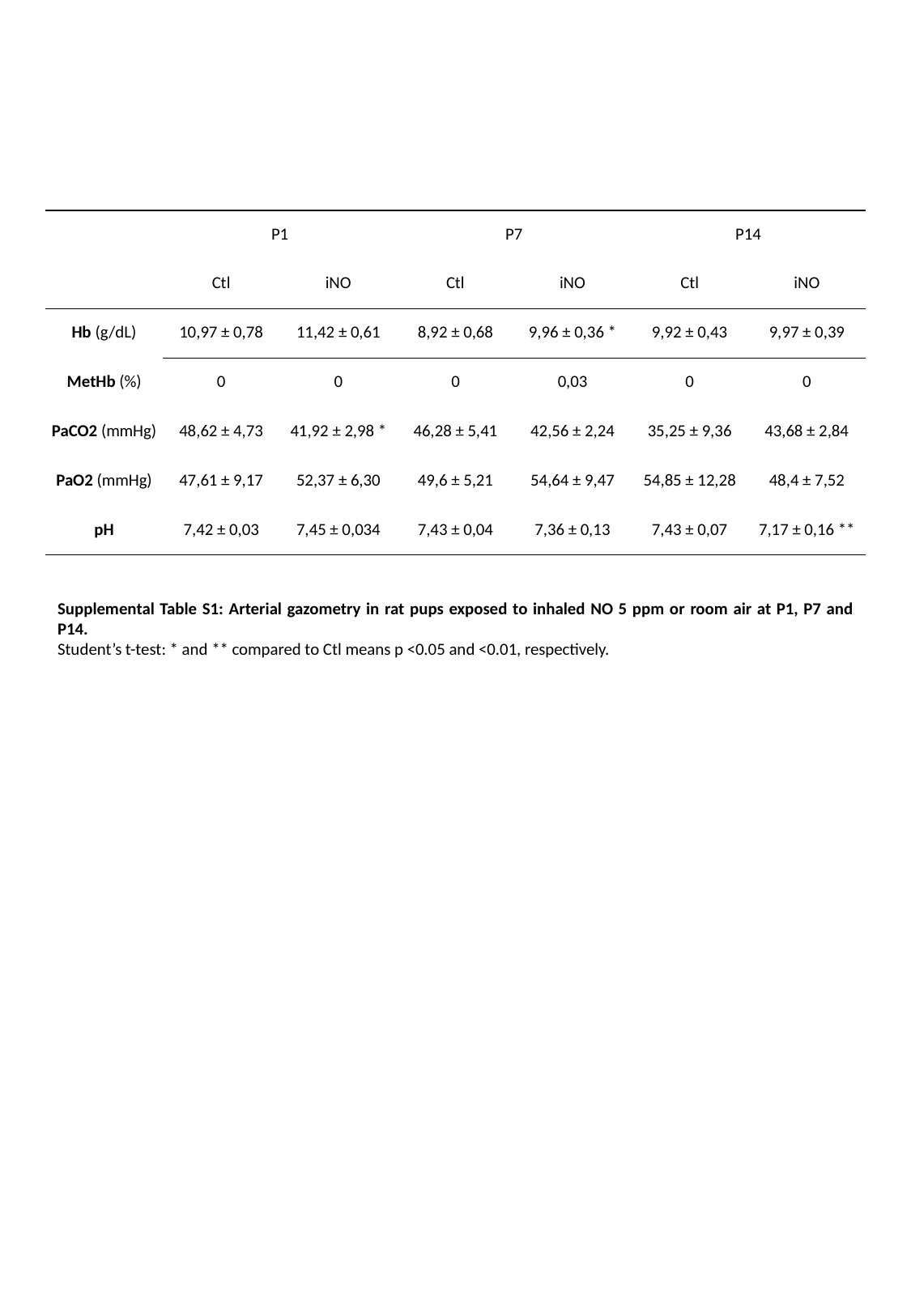

| | P1 | | P7 | | P14 | |
| --- | --- | --- | --- | --- | --- | --- |
| | Ctl | iNO | Ctl | iNO | Ctl | iNO |
| Hb (g/dL) | 10,97 ± 0,78 | 11,42 ± 0,61 | 8,92 ± 0,68 | 9,96 ± 0,36 \* | 9,92 ± 0,43 | 9,97 ± 0,39 |
| MetHb (%) | 0 | 0 | 0 | 0,03 | 0 | 0 |
| PaCO2 (mmHg) | 48,62 ± 4,73 | 41,92 ± 2,98 \* | 46,28 ± 5,41 | 42,56 ± 2,24 | 35,25 ± 9,36 | 43,68 ± 2,84 |
| PaO2 (mmHg) | 47,61 ± 9,17 | 52,37 ± 6,30 | 49,6 ± 5,21 | 54,64 ± 9,47 | 54,85 ± 12,28 | 48,4 ± 7,52 |
| pH | 7,42 ± 0,03 | 7,45 ± 0,034 | 7,43 ± 0,04 | 7,36 ± 0,13 | 7,43 ± 0,07 | 7,17 ± 0,16 \*\* |
Supplemental Table S1: Arterial gazometry in rat pups exposed to inhaled NO 5 ppm or room air at P1, P7 and P14.
Student’s t-test: * and ** compared to Ctl means p <0.05 and <0.01, respectively.

## Slide 2
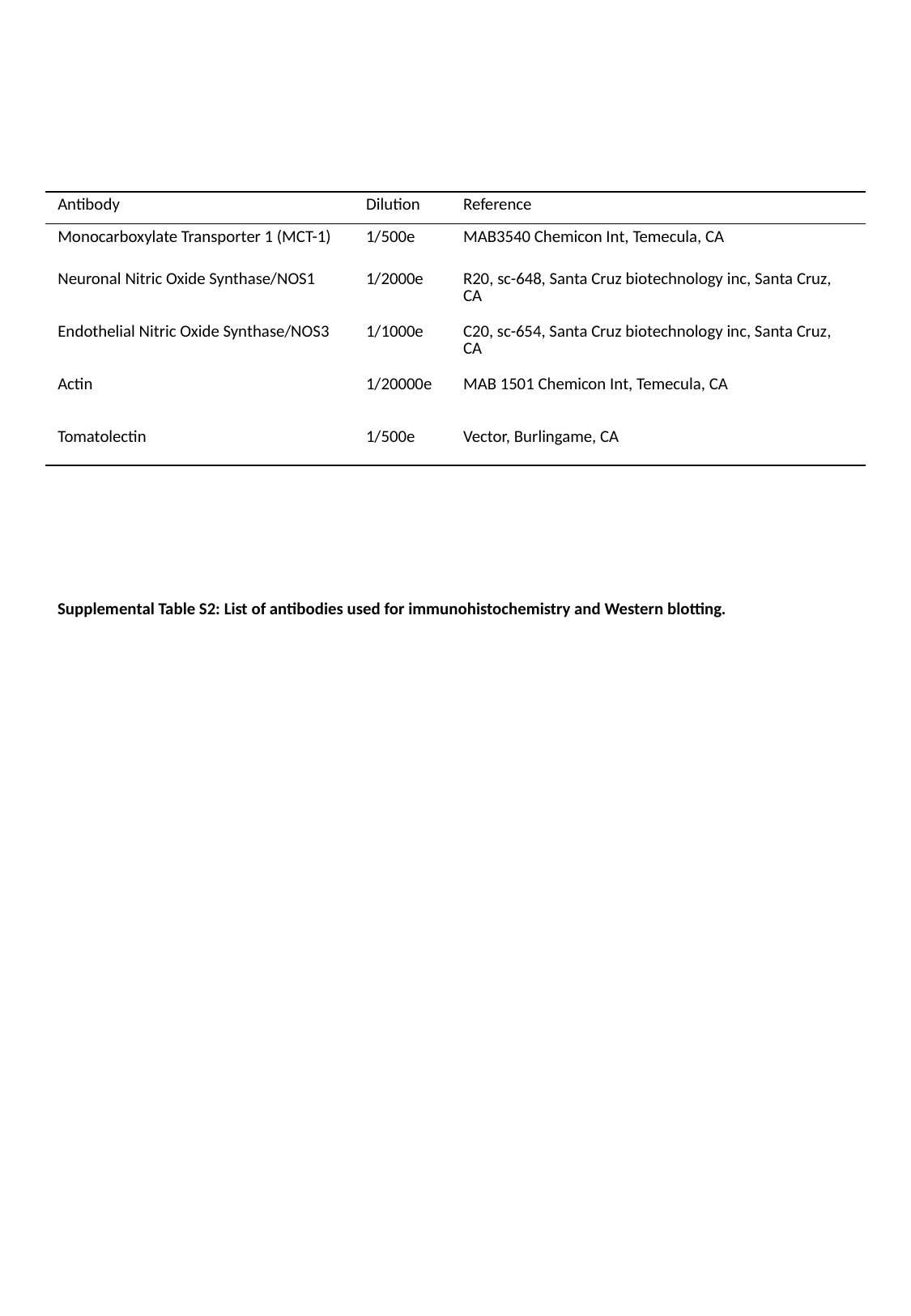

| Antibody | Dilution | Reference |
| --- | --- | --- |
| Monocarboxylate Transporter 1 (MCT-1) | 1/500e | MAB3540 Chemicon Int, Temecula, CA |
| Neuronal Nitric Oxide Synthase/NOS1 | 1/2000e | R20, sc-648, Santa Cruz biotechnology inc, Santa Cruz, CA |
| Endothelial Nitric Oxide Synthase/NOS3 | 1/1000e | C20, sc-654, Santa Cruz biotechnology inc, Santa Cruz, CA |
| Actin | 1/20000e | MAB 1501 Chemicon Int, Temecula, CA |
| Tomatolectin | 1/500e | Vector, Burlingame, CA |
Supplemental Table S2: List of antibodies used for immunohistochemistry and Western blotting.

## Slide 3
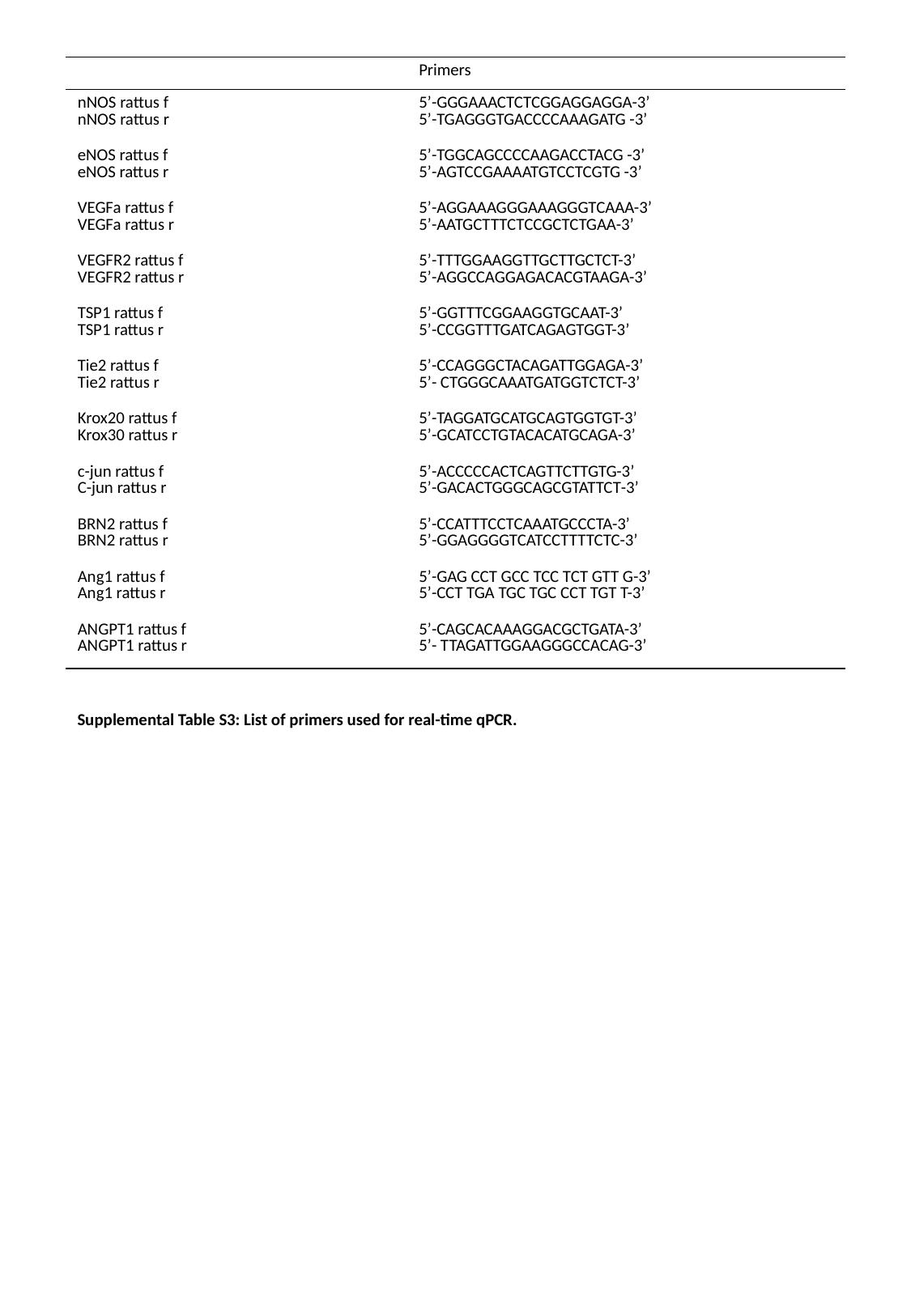

| | Primers |
| --- | --- |
| nNOS rattus f nNOS rattus r | 5’-GGGAAACTCTCGGAGGAGGA-3’ 5’-TGAGGGTGACCCCAAAGATG -3’ |
| eNOS rattus f eNOS rattus r | 5’-TGGCAGCCCCAAGACCTACG -3’ 5’-AGTCCGAAAATGTCCTCGTG -3’ |
| VEGFa rattus f VEGFa rattus r | 5’-AGGAAAGGGAAAGGGTCAAA-3’ 5’-AATGCTTTCTCCGCTCTGAA-3’ |
| VEGFR2 rattus f VEGFR2 rattus r | 5’-TTTGGAAGGTTGCTTGCTCT-3’ 5’-AGGCCAGGAGACACGTAAGA-3’ |
| TSP1 rattus f TSP1 rattus r | 5’-GGTTTCGGAAGGTGCAAT-3’ 5’-CCGGTTTGATCAGAGTGGT-3’ |
| Tie2 rattus f Tie2 rattus r | 5’-CCAGGGCTACAGATTGGAGA-3’ 5’- CTGGGCAAATGATGGTCTCT-3’ |
| Krox20 rattus f Krox30 rattus r | 5’-TAGGATGCATGCAGTGGTGT-3’ 5’-GCATCCTGTACACATGCAGA-3’ |
| c-jun rattus f C-jun rattus r | 5’-ACCCCCACTCAGTTCTTGTG-3’ 5’-GACACTGGGCAGCGTATTCT-3’ |
| BRN2 rattus f BRN2 rattus r | 5’-CCATTTCCTCAAATGCCCTA-3’ 5’-GGAGGGGTCATCCTTTTCTC-3’ |
| Ang1 rattus f Ang1 rattus r | 5’-GAG CCT GCC TCC TCT GTT G-3’ 5’-CCT TGA TGC TGC CCT TGT T-3’ |
| ANGPT1 rattus f ANGPT1 rattus r | 5’-CAGCACAAAGGACGCTGATA-3’ 5’- TTAGATTGGAAGGGCCACAG-3’ |
Supplemental Table S3: List of primers used for real-time qPCR.

## Slide 4
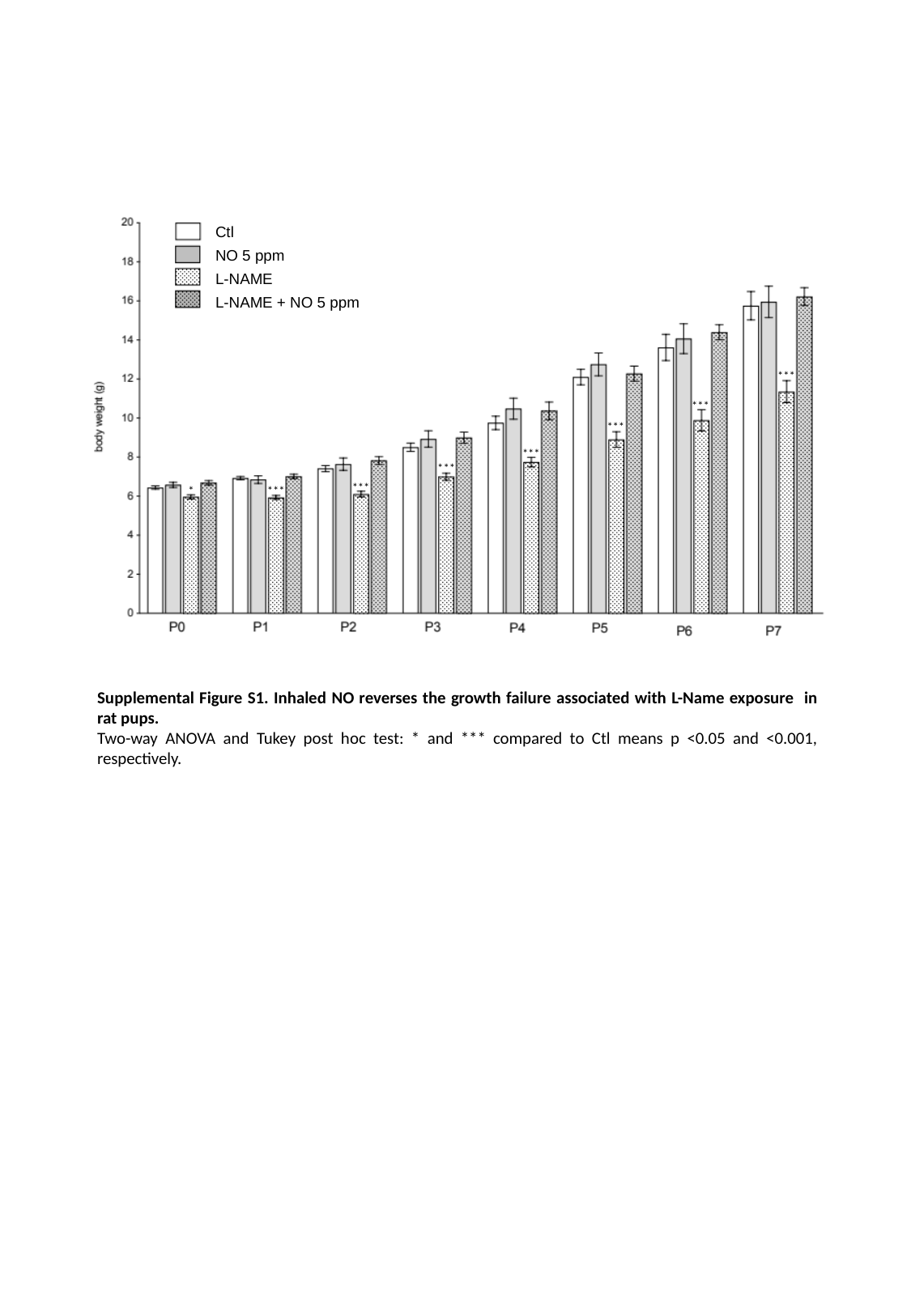

Ctl
NO 5 ppm
L-NAME
L-NAME + NO 5 ppm
***
***
***
***
***
***
*
***
Supplemental Figure S1. Inhaled NO reverses the growth failure associated with L-Name exposure in rat pups.
Two-way ANOVA and Tukey post hoc test: * and *** compared to Ctl means p <0.05 and <0.001, respectively.

## Slide 5
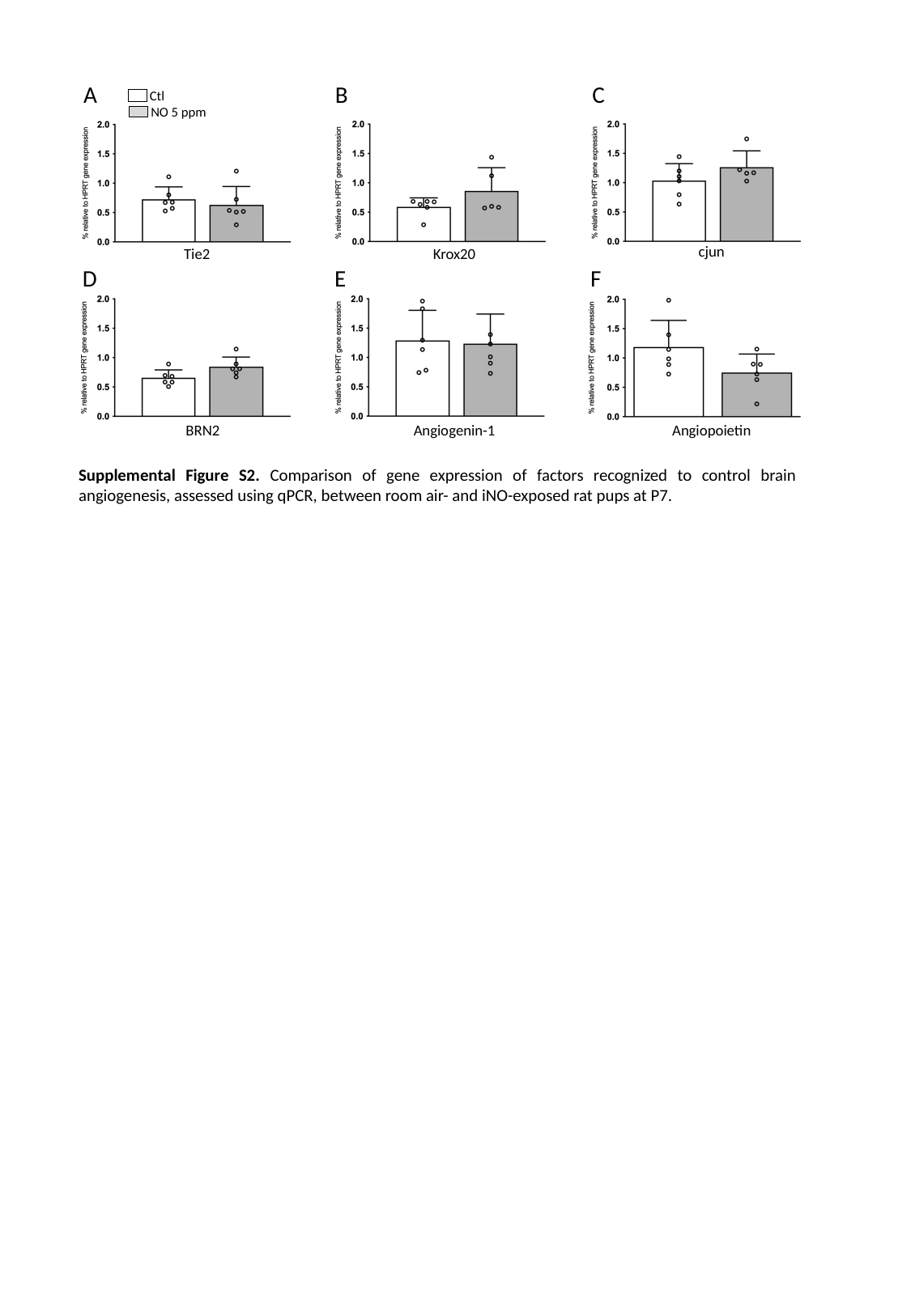

B
C
A
Ctl
NO 5 ppm
cjun
Tie2
Krox20
D
F
E
BRN2
Angiogenin-1
Angiopoietin
Supplemental Figure S2. Comparison of gene expression of factors recognized to control brain angiogenesis, assessed using qPCR, between room air- and iNO-exposed rat pups at P7.

## Slide 6
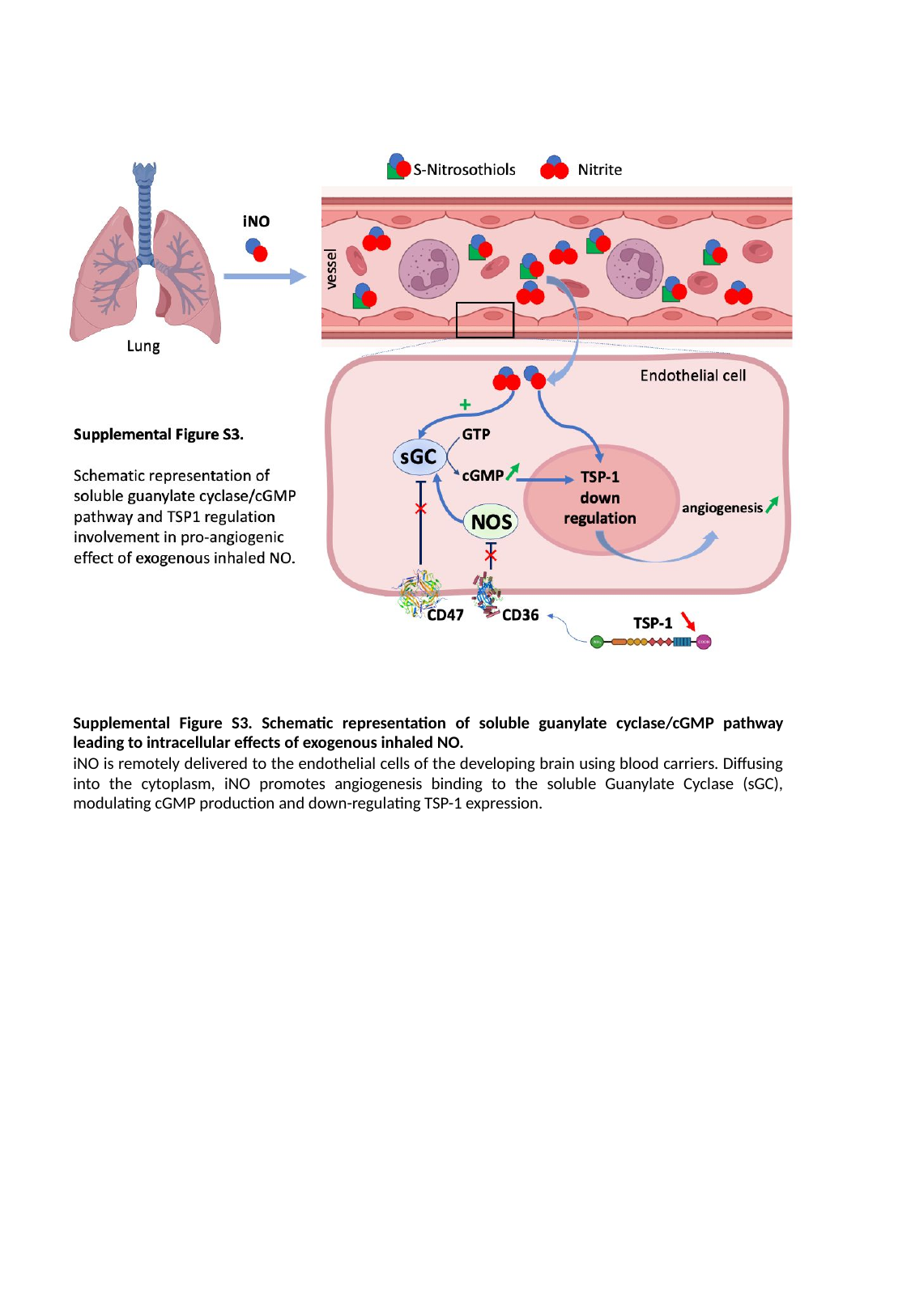

Supplemental Figure S3. Schematic representation of soluble guanylate cyclase/cGMP pathway leading to intracellular effects of exogenous inhaled NO.
iNO is remotely delivered to the endothelial cells of the developing brain using blood carriers. Diffusing into the cytoplasm, iNO promotes angiogenesis binding to the soluble Guanylate Cyclase (sGC), modulating cGMP production and down-regulating TSP-1 expression.
